# Supplementary material for: Original speech and its echo are segregated and separately processed in the human brain
Source: PLoS Biol. 2024 Feb 15;22(2):e3002498. doi: 10.1371/journal.pbio.3002498 (PMC10868781; doi:10.1371/journal.pbio.3002498)
Supplement: S1 Text — Simulations of a depression model and an optimal filter model. (DOCX) [file pbio.3002498.s001.docx]

**Supporting methods**

**Simulation of a synaptic depression model**

The input to the synaptic depression model [1], i.e., presynaptic activity, was the auditory spectrogram *s*(*t*, *c*), which contained 128 frequency channels and each frequency channel was independently processed by the depression model. In the following, the level of depression was denoted as *d*(*t*, *c*) for frequency channel *c* and time bin *t*. It was bounded between 0 and 1, and was initialized at 0 for *t* = 1. Its dynamics were described as:

$$\begin{aligned} \text{ d}\text{(}\text{t}\text{, }\text{c}\text{) }\text{= }\text{d}\text{(}\text{t }\text{- 1, }\text{c}\text{) }\text{+ }\text{s}\text{(}\text{t }\text{- 1, }\text{c}\text{)(1 - }\text{d}\text{(}\text{t }\text{- 1, }\text{c}\text{))}\text{v}\text{ - }\frac{\text{d}\text{(}\text{t}\text{- 1, }\text{c}\text{)}}{\text{τ}} \text{,}\#(1) \end{aligned}$$

where *v* and *τ* controlled the strength of depression and the time constant of recovery. To find the parameters that could best explain the neural response coherence spectrum, we scanned *v* in {0.05, 0.1, 0.2, 0.3, 0.4}/max(*s*(*t*, *c*)), and scanned *τ* in {50, 100, 250, 500, 1,000, 1,500} ms. The postsynaptic response *r_d_* (*t*, *c*) was computed as follows:

$$\begin{aligned} \text{ r}\text{d}\text{(}\text{t}\text{, }\text{c}\text{) = }\text{y}\text{(}\text{t}\text{, }\text{c}\text{)(1 - }\text{d}\text{ (}\text{t}\text{, }\text{c}\text{)) }\text{,}\#\left( 2 \right) \end{aligned}$$

the simulated depressed neural response *r*(*t*), was obtained by summing the “depressed” spectrogram over frequency channels.

**Simulation of an optimal filter model**

We simulated whether an optimal linear filter [2] could effectively cancel the effects of echoes. The optimal filter TRF*_o_* was designed by solving the following equation using ridge regression:

$$\begin{aligned} \text{ s}_{\text{d}}\text{(}\text{t}\text{) }\text{=}\sum_{\text{τ}\text{ }\text{= 1}}^{\text{D}} \text{TRF}_{\text{o}}\text{(}\text{τ}\text{)}\text{s}\text{(}\text{t }\text{- }\text{τ}\text{) + }\text{e}\text{(}\text{t}\text{)} \text{,}\#\left( 3 \right) \end{aligned}$$

where *s*(*t*), *s_d_*(*t*), and *e*(*t*) denoted the envelope of echoic speech, the envelope of direct sound and the residual error, respectively. The output of the optimal filter, i.e., the simulated neural response, was:

$$\begin{aligned} \text{ }\text{r}\text{(}\text{t}\text{) }\text{=}\sum_{\text{τ}\text{ }\text{= 1}}^{\text{D}} \text{TRF}_{\text{o}}\text{(}\text{τ}\text{)}\text{s}\text{(}\text{t }\text{- }\text{τ}\text{) .}\#\left( 4 \right) \end{aligned}$$

The optimal filter was not expected to completely remove the echo effect for the following reason. Adding an echo to a signal was equivalent to filtering the signal using a linear filter, and the transfer function of the linear filter corresponding to the echoic speech in the current study was *H*(*z*) = *A* + *z ^– N^*, where *A* was the amplitude of the direct sound and *N* was the delay of echo in terms of the number of samples. In the current study, *A* was 1 or 0.5, rendering the transfer function irreversible. In other words, the echoes in the current study could not be completely cancelled by any linear filters.

**References**

1. David SV, Mesgarani N, Fritz JB, Shamma SA. Rapid Synaptic Depression Explains Nonlinear Modulation of Spectro-Temporal Tuning in Primary Auditory Cortex by Natural Stimuli. Journal of Neuroscience. 2009;29: 3374–3386. doi:10.1523/JNEUROSCI.5249-08.2009

2. Ivanov AZ, King AJ, Willmore BD, Walker KM, Harper NS. Cortical adaptation to sound reverberation. Bathellier B, Shinn-Cunningham BG, Bathellier B, Mesgarani N, editors. eLife. 2022;11: e75090. doi:10.7554/eLife.75090
