# Supplementary figures and images for: Original speech and its echo are segregated and separately processed in the human brain
Source: PLoS Biol. 2024 Feb 15;22(2):e3002498. doi: 10.1371/journal.pbio.3002498 (PMC10868781; doi:10.1371/journal.pbio.3002498)

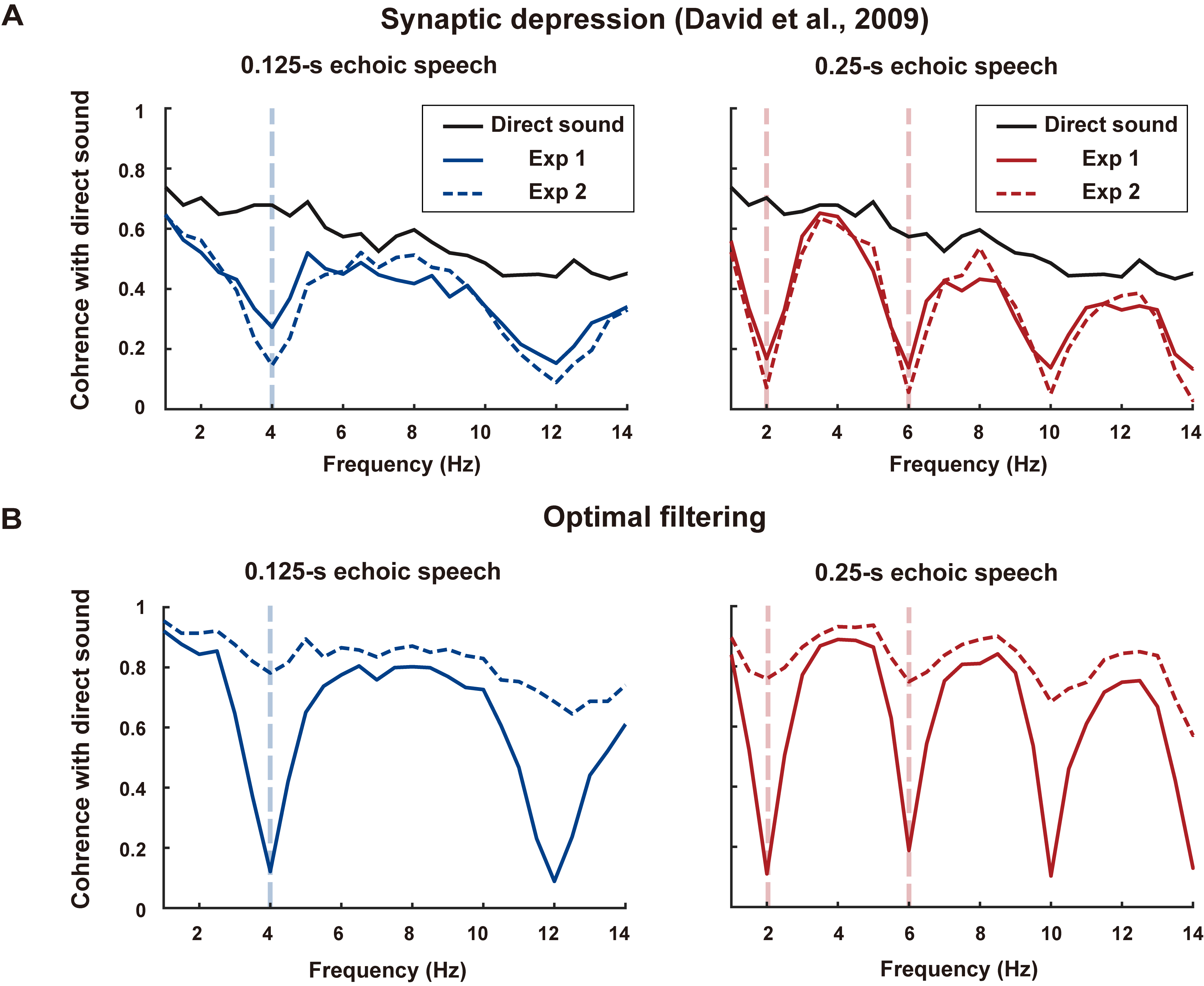

Supplement: S1 Fig — (A) Neural responses are simulated by processing the speech envelope using the synaptic depression model in David and colleagues (2009). (B) Neural responses are simulated by processing the speech envelope using the optimal adaptive filter similar to that of Ivanov and colleagues (2022). The underlying data can be found at https://zenodo.org/records/10472483. (TIF) [file pbio.3002498.s002.tif]

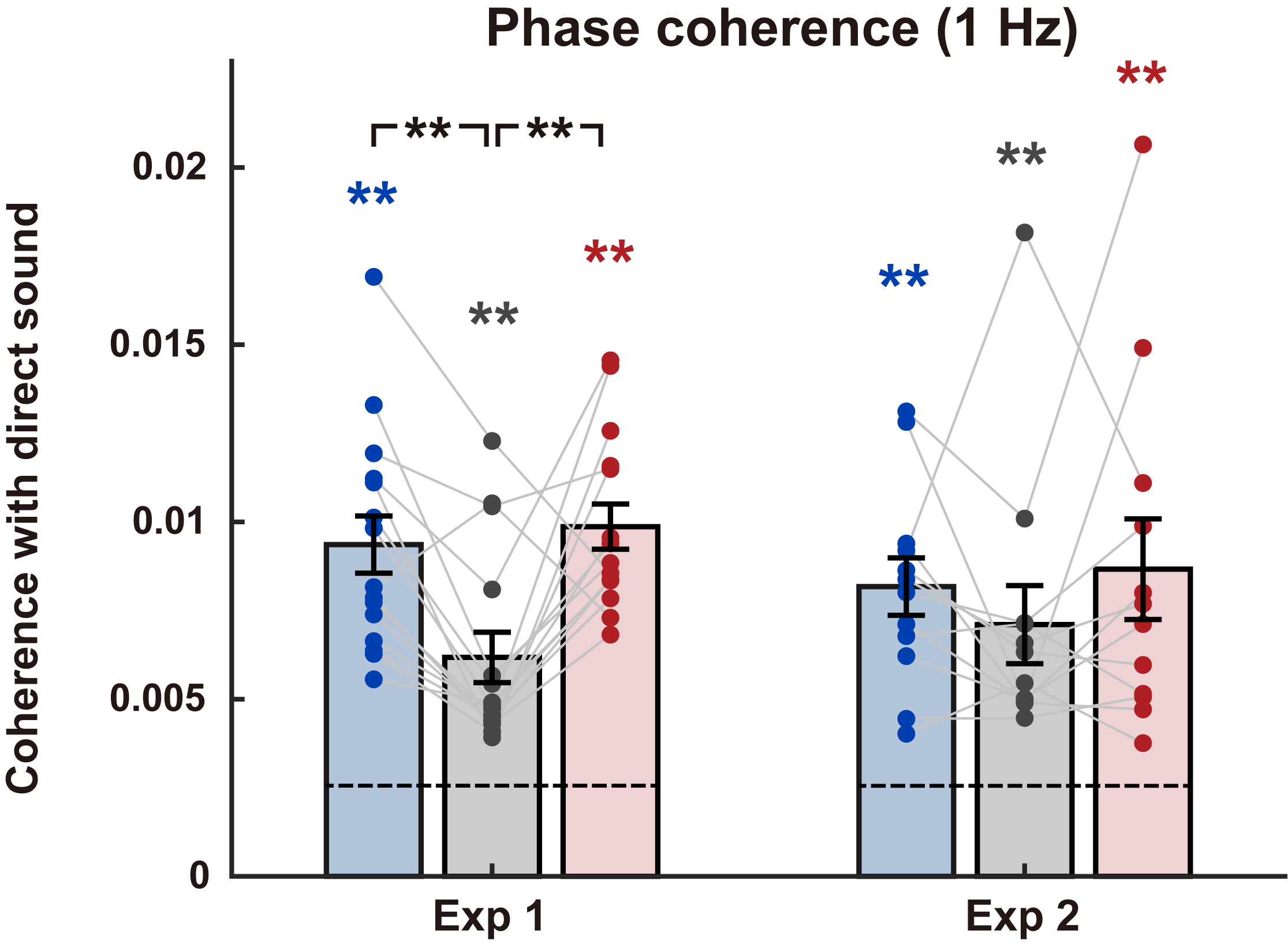

Supplement: S2 Fig — Each dot represents 1 individual and error bars represent 1 SEM. Dashed black lines show chance-level phase coherence. Phase coherence significantly higher than chance level and significant differences between conditions are marked (* p < 0.05, ** p < 0.01, permutation test, FDR corrected). The underlying data can be found at https://zenodo.org/records/10472483. (TIF) [file pbio.3002498.s003.tif]

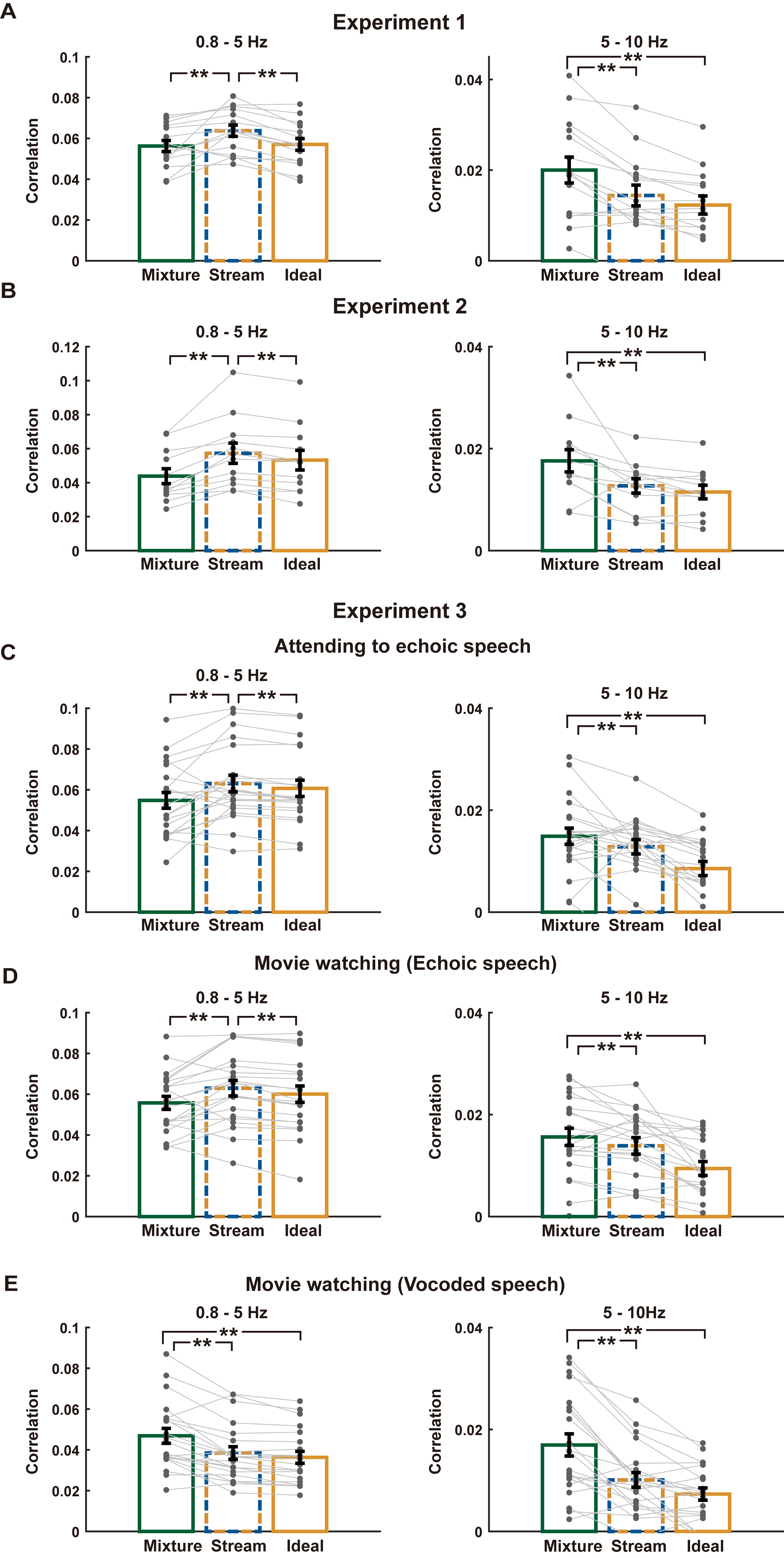

Supplement: S3 Fig — Predictive powers of the TRF models in 0.8–5 Hz (left plots) and 5–10 Hz (right plots), averaged over participants and MEG gradiometers. Gray dots show individual participants. Error bars represent 1 SEM across participants. The underlying data can be found at https://zenodo.org/records/10472483. (TIF) [file pbio.3002498.s004.tif]

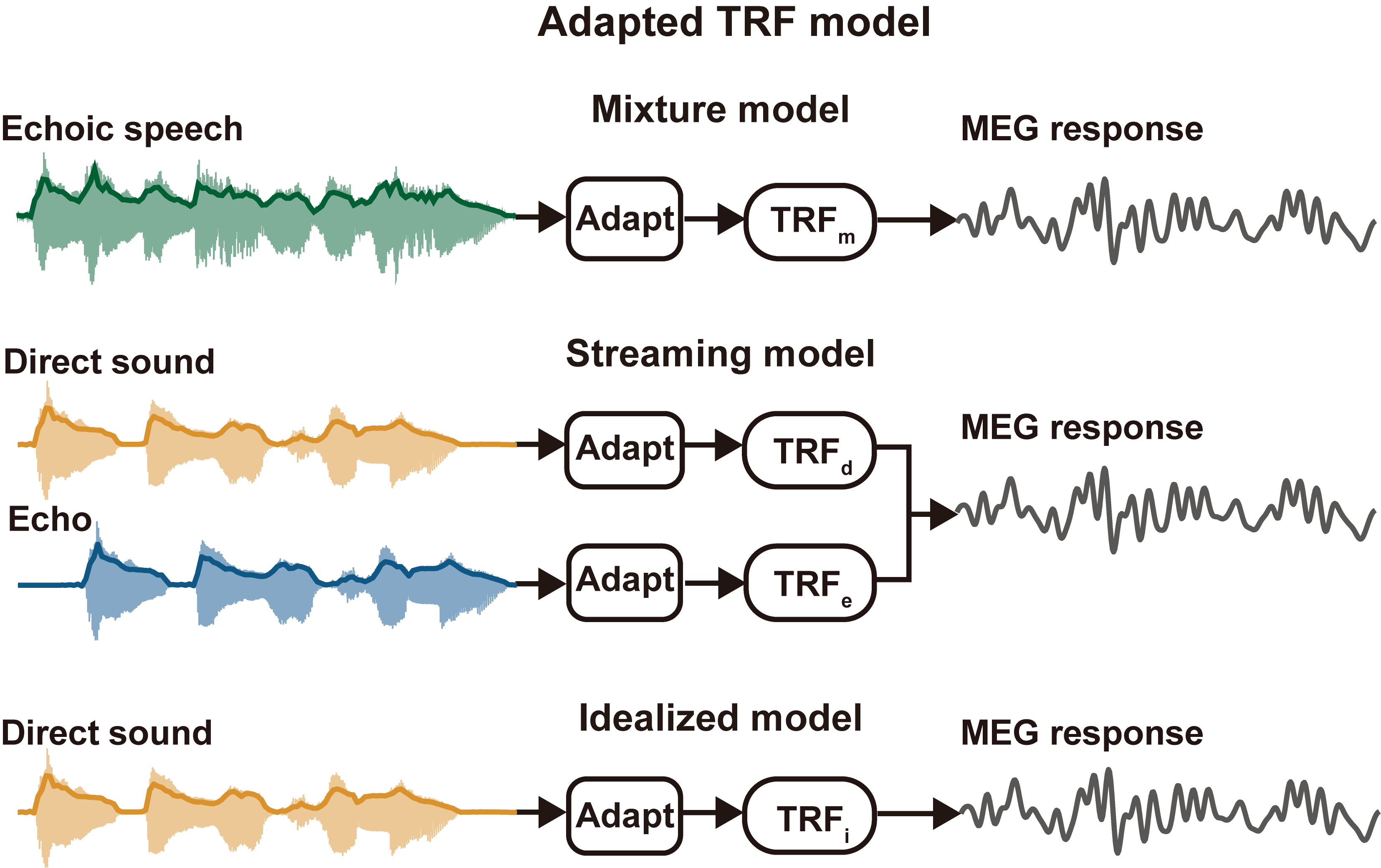

Supplement: S4 Fig — (TIF) [file pbio.3002498.s005.tif]

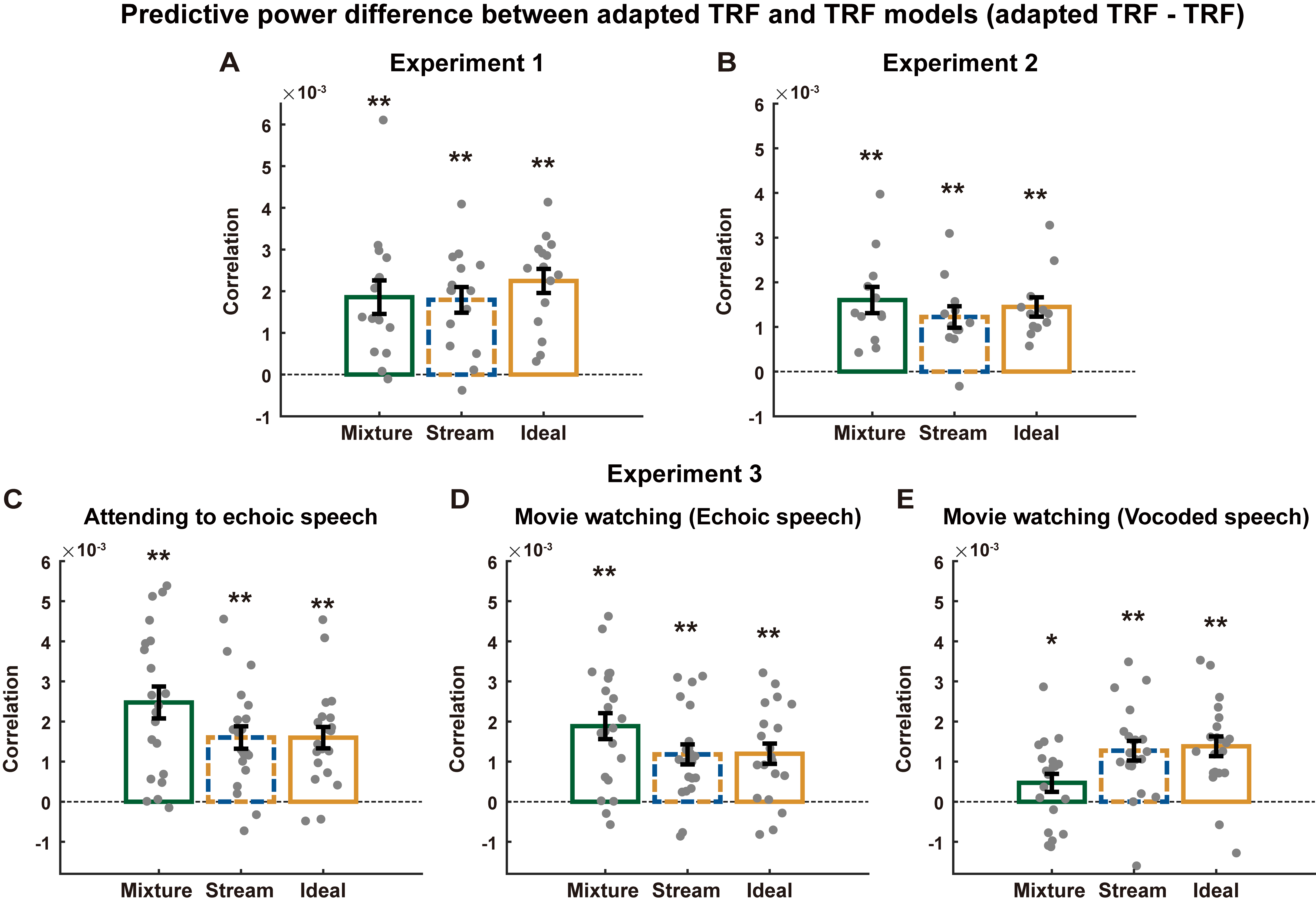

Supplement: S5 Fig — The difference between the predictive powers of adapted TRF and TRF model, averaged over participants and MEG gradiometers. Gray dots show individual participants. Error bars represent 1 SEM across participants. The underlying data can be found at https://zenodo.org/records/10472483. (TIF) [file pbio.3002498.s006.tif]
