# Supplementary material for: Original speech and its echo are segregated and separately processed in the human brain
Source: PLoS Biol. 2024 Feb 15;22(2):e3002498. doi: 10.1371/journal.pbio.3002498 (PMC10868781; doi:10.1371/journal.pbio.3002498)
Supplement: Attachment — Submitted filename: Response to Reviewers-PBIOLOGY.pdf [file pbio.3002498.s010.pdf]

Reviewer #1:

This study examines neural (MEG) responses to echoic speech, i.e. speech that is mixed with a delayed version of itself. It is inspired by the fact that we can understand such speech quite well as evidenced by the current era of zoom calls as well as previous studies from the same group. This observation implies that original speech is perceptually segregated from its echo.

The authors claim to show that, when exposed to echoic speech, MEG responses track the slow temporal modulations (< 10 Hz) that were present in original speech but reduced in the stimulus by the addition of the echo. They also show results from temporal response function (TRF) models that evidence a neural segregation into original speech and echo.

I am sympathetic with the authors' approach and find the topic interesting and certainly relevant for research on auditory scene analysis. However, I am currently not convinced that their results support several of the main claims (including that in the title). This might be partly due to me not fully understanding the analytical approach and I would be grateful for some clarifications.

Thank you for the accurate summary and positive evaluation.

This mostly concerns what is shown in Fig. 1C, D and the corresponding results in Fig. 2. If I understand the rationale correctly, the envelope of the original speech includes power around 4 Hz (?) which is reduced by the addition of the echo. If the brain only tracked the incoming signal, then tracking at 4 Hz should therefore be reduced in the echoic speech condition. However, if it segregates original speech and echo, then we should find 4 Hz tracking even in the echoic condition, as a reflection of stream segregation. If my summary is incorrect, then I would be grateful for more details on the study's aim. If it is correct, I do not understand why the authors do not show the envelope spectrum of the original speech, extract the dominant frequency (such as 4 Hz), and then test whether tracking of this frequency (e.g., phase coherence between MEG and speech envelope) is preserved in the echoic condition. An envelope spectrum for the echoic speech would also be helpful. If there is a peak in the tracking response to echoic speech that corresponds to one in the envelope spectrum for original but not echoic speech, I would be convinced that the temporal modulation is restored as claimed.

Thank you for the helpful suggestion. We have now shown the modulation spectra in Figs. 1B, C. The modulation spectra analysis clearly revealed that an echo attenuates the temporal envelope at echo-related frequencies (i.e., 4Hz for 0.125-s echoic speech, 2 and 6Hz for 0.25-s echoic speech). We have also added an illustration about why these frequencies are affected by the echo in Fig. 1A.

"A modulation spectrum analysis clearly revealed that an echo could notch out the temporal modulations at some frequencies that were related to the echo delay (Fig

1B and 1C). Fig 1A illustrates why these notches were created – The Fourier analysis decomposed a signal into sinusoids. If the signal was time shifted by T, forming an echo that had the same amplitude as the direct sound, all its Fourier components would also be shifted by T. Consider a sinusoidal Fourier component whose period was 2T, which had an opposite phase for the original signal and the echo, would get cancelled when the original signal and the echo were mixed. The same applied to Fourier components whose periods were 2T/3, 2T/5, etc. In the following, the frequencies of these sinusoidal components that were notched out by the echo, i.e., 1/2T, 3/2T, 5/2T, were referred to as the echo-related frequencies."

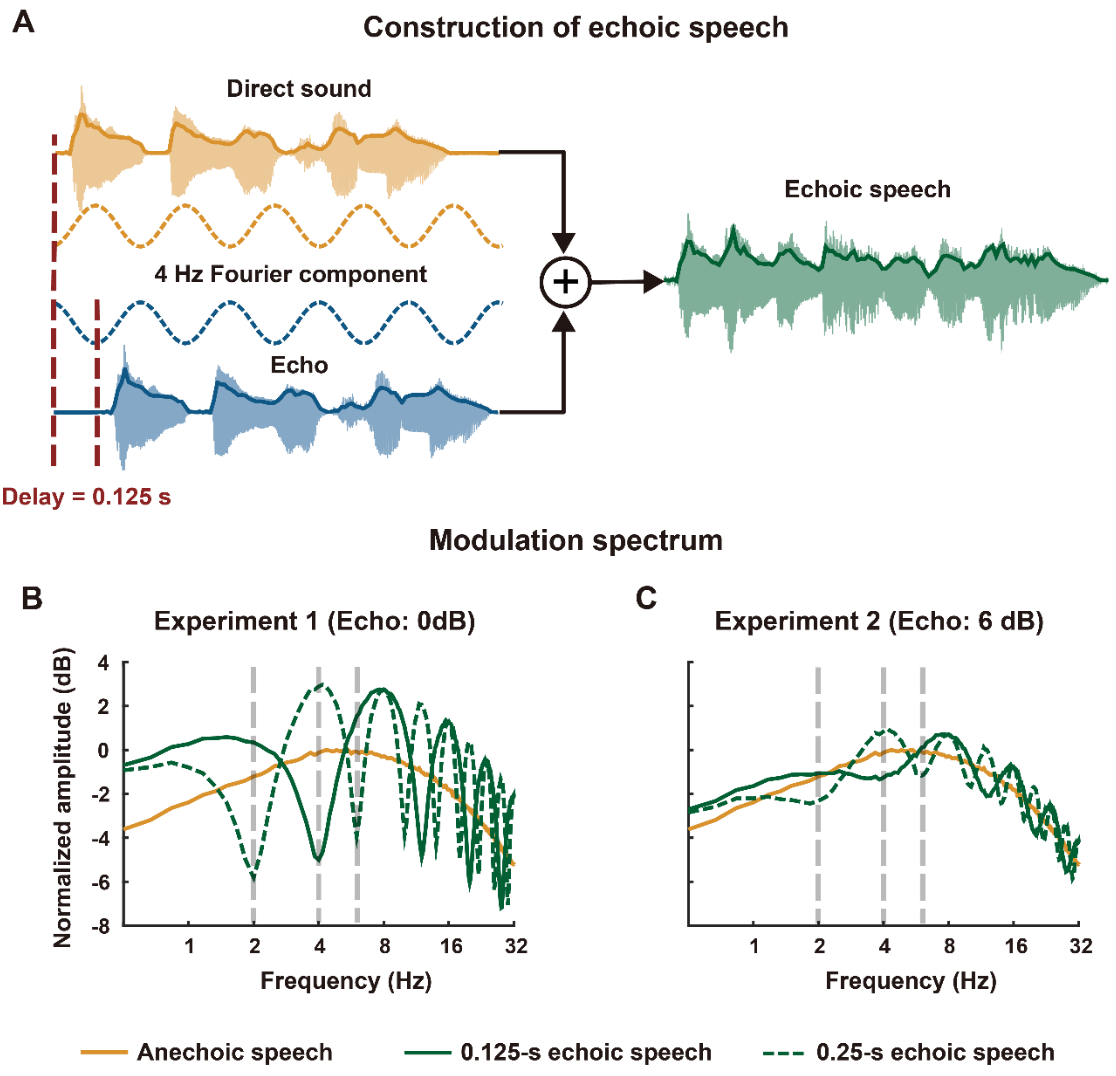

"Fig 1. Construction of echoic speech and the modulation spectrum.

(A) In Experiment 1, the echoic speech is generated by delaying a speech signal by T (0.125 s or 0.25 s) and adding together the delayed signal with the original signal. The dashed sinusoids in yellow and blue represent the 1/2T Hz Fourier components of the envelope of direct sound and echo, respectively. The two sinusoids are 180 degrees out of phase and are fully cancelled in the mixture. (B) Modulation spectrum

of stimulus in Experiment 1. Dashed grey lines denote the echo-related frequencies below 10 Hz. (C) Modulation spectrum of stimulus in Experiment 2, in which the amplitude of the delayed signal is twice the amplitude of the direct sound (6-dB echo).”

I agree that conventional analyses of speech tracking are often based on phase-locking between neural signal and speech, but it is not fully clear to me how this is linked to the phase coherence between original and echoic speech, and the focus on notches. If the brain does not segregate original speech and echo, I would still expect to see reduced tracking as a result from reduced power of temporal modulations in the echoic stimulus. Similarly, the term "echo-related frequencies" was not clear to me (why are they echo related? Is there power in the echo at those frequencies?).

Sorry for the confusion. The term “echo-related frequencies” are now more clearly defined and the underlying reason is illustrated (see the response to the previous question). We have now also clarified the rationale to calculate the phase coherence between the temporal envelope of echoic speech and the temporal envelope of the direct sound. In brief, we used the temporal envelope of echoic speech as a simulation of neural activity that only tracks the temporal envelope of echoic speech.

“Our hypothesis, referred to as the envelope restoration hypothesis, was that the auditory system could fully or partially restore the temporal envelope of direct sound, and an alternative hypothesis was that the auditory system faithfully followed the temporal envelope of the echoic speech. We first quantified the prediction of the alternative hypothesis through a simulation, in which the neural response was simply simulated using the envelope of echoic speech. The phase coherence spectrum between the simulated neural response and the envelope of direct sound showed notches at 4 and 12 Hz for speech with a 0.125-s echo, and showed notches at 2, 6, 10, and 14 Hz for speech with a 0.25-s echo (Fig 2A). These results demonstrated that if neural activity faithfully tracked the envelope of echoic speech, it would show very low phase coherence (< 0.07) with the envelope of the direct sound at echo-related frequencies. Therefore, if the phase coherence between the neural response to echoic speech and the temporal envelope of direct sound is near 0 at echo-related frequencies, the alternative hypothesis is supported. Otherwise, the envelope restoration hypothesis is supported and full restoration is suggested if the phase coherence value is comparable to the neural responses to echoic speech and direct sound.”

Other, including minor comments (random order):

1. I wonder whether the term "automatic" is really necessary as this is hard to demonstrate.

We agree that the term is not necessary. We have removed this term and only kept a discussion about how attention modulates auditory streaming.

2. At some point the nomenclature changes from "direct speech" to "anechoic speech", this was a bit confusing and it would be good to clarify that both refer to the same stimulus.

We have made sure that now the "direct sound" refers to the leading sound in echoic speech and "anechoic speech" refers to speech that is not mixed with an echo. We have also clearly mentioned that they are the same.

"In the anechoic speech condition, listeners only listened to the direct sound without being accompanied by an echo."

3. If the aim is to compare tracking at certain frequencies across conditions, it might be important to correct for differences in 1/f slopes that are visible in Fig. 2.

Thank you for raising the very important point. On the one hand, we have made it clear that a primary hypothesis to test is whether the phase coherence between MEG response and the envelope of direct sound is above chance, and this hypothesis does not involve a comparison between conditions (see the reply to the major concern 2). On the other hand, we do have several comparisons across conditions. In the previous analysis, we only focused on the echo-related frequencies. However, as the reviewer correctly pointed out, the phase coherence also showed differences across conditions at very low frequencies, and we have now added discussions about these differences (see below, which benefited from the modulation spectrum analysis suggested by the reviewer). In general, the 1/f trend in the phase coherence spectrum is different from the 1/f trend in the power spectrum. The 1/f trend in the power spectrum can reflect spontaneous neural activity not phase locked to speech, and the power of spontaneous neural activity may linearly add to the power of speech-tracking activity. Therefore, removing the 1/f trend in the power spectrum can lead to a better estimate of the speech-tracking response. The phase coherence spectrum, however, captures the phase locking between neural activity and speech, and the 1/f trend in the phase coherence reflects that the auditory cortex can better phase lock to slower temporal modulations. Therefore, we chose to keep this trend in the data analysis.

Discussion on the difference between very low-frequency phase coherence across conditions:

"Here, in Experiments 1 and 2, the phase coherence below 2 Hz was generally enhanced for echoic speech than anechoic speech (Fig 2B and 3C, S2 Fig), which might be explained by the increase in sound intensity for echoic speech compared with anechoic speech at low frequencies, as shown in the modulation spectrum (Fig 1B and 1C)."

Reviewer #2:

The ms. tests the possible mechanisms of the previously observed excellent comprehension of echoed speech. The results of two experiments show that adaptation models do not accurately explain the correspondence between magnetic brain activity and the speech envelope. Modeling the results of the third experiment shows that a general linear model based on the assumption that the original speech and its echo are segregated and separately processed in the brain better predict the observed magnetic brain activity than similar models based either on the assumption that the full signal (original speech + echo) or only the original speech signal is processed as the input of the model. The paper thus suggests that in contrast to how reverberation is handled by the brain, echoes are processed with the help of first segregating them from the original speech signal.

This is an excellent ms., using computational modeling to decide about the likely algorithms the brain uses to extract meaning from echoed speech. The study is well-motivated, and the relevant literature is cited. The experiments are mostly well designed and address the important aspects of the questions of the ms.; measurements and signal processing are clear and to the point (see some minor comments, though). Thus, the results are reliable and support the conclusions of the ms.

Thank you for the accurate summary and positive evaluation.

The following comments are meant to improve the readability and impact of the ms.
Major point:

The study consists of two parts. The first part shows the problems adaptation models encounter in explaining the correspondence between the signal of the echoed speech segment and the envelope of the concurrent magnetic brain activity (Experiments I and II). The second part then tests whether general linear models better explain the observed brain activity if they assume that the original speech and its echo have been segregated prior to entering the modeled processing compared to entering the composite or the original (unechoed) signal.

There are some issues with the second part, which are omitted or not discussed in the ms.

1. It is implicitly assumed that auditory stream segregation precedes the processes resulting in speech envelope tracking. This is quite likely not the case. The authors themselves argue that auditory stream segregation is helped by low level cues (which include the dynamic tracking of speech energy) as well as high-level ones (which include comprehending the speech stream so far). This makes it very likely that speech processing (including echo processing) goes hand-in-hand with auditory stream segregation.

Thank you for bringing up this very important issue. We fully agree that speech processing goes hand-in-hand with auditory stream segregation. In fact, the concern raised here also apply to previous studies that investigate speech

segregation using MEG or similar techniques. We have added discussions on this point.

"Here, the results suggest that neural activity separately tracks the temporal envelope of the direct sound and the temporal envelope of the echo. The temporal envelope of a sound, however, is extracted even in the auditory periphery, while auditory stream segregation occurs in much later auditory processing stages [3,16,24,48,49], leading to an apparent paradox of why auditory streaming can modulate envelope-tracking activity. A solution to the paradox is the following: What is available in the auditory periphery is the temporal envelope of the mixture, while the temporal envelope of the direct sound or the echo can only be resolved after auditory stream segregation. For example, under the analysis-by-synthesis framework [3,4], the speech mixture is first decomposed into features and features belonging to an auditory stream are selectively grouped together to form a representation of that stream in later processing stages. In other words, the temporal envelope of the sound mixture is extracted in the cochlear while the temporal envelope of an auditory stream is resynthesized in cortex [13,16,50]. Given the relatively low spatial resolution of MEG and the lack of structural MRI scans from the participants, here we did not investigate where anatomically the temporal envelope of the direct sound is resynthesized. Future studies, possibly requiring intracranial neural recordings from humans or animal neurophysiology, are required to analyze where the segregation between speech and echo emerges along the auditory pathway."

2. Second, it is quite unlikely that the output of auditory stream segregation is a restored sound signal, which could be fed into to further processing (including the processing of echoes). Thus, the related point needs to be made much more carefully (including the abstract and the conclusions), and limitations of this approach should be spelled out.

Thank you for the very constructive suggestion. In the current study, auditory stream segregation is considered as a mechanism to separately process features belonging to the direct sound and the echo. We have made this point clear by adding the following sentence to the first paragraph of Discussion.

"Here, the key difference between the auditory stream segregation and lower-level neural adaptation mechanisms was that auditory stream segregation allowed differential processing of features belonging to the direct sound and the echo."

In the abstract, we now concluded that:

"These results strongly suggested that, through mechanisms such as stream segregation, the auditory system can build an echo-insensitive representation of speech envelope, which can support reliable speech recognition."

3. The paper is aimed to make the point that assuming segregation of the echo from the original speech is a more likely approach of the brain to the problem of echoes than neural adaptation. However, the two are never directly compared. Thus, it should be discussed how well the models compared in the second part cover adaptation or test how well adaptation models would work if stream segregation preceded the adaptation phenomena. In fact, adaptation might not be directly comparable to stream segregation, because while stream segregation is a high-level process, adaptation is often treated as a low-level mechanism. (Or spell out a high-level version of adaptation to be contrasted with stream segregation, if this is how you wish to consider adaptation.) Thus, it is quite possible that there is no real contradiction between the two, as they represent different levels of explanation. These issues should be carefully discussed, else the two parts of the ms. fall apart.

Thank you for bringing up this very important issue. Indeed, neural adaptation does not contradict with auditory stream segregation. Neural adaptation always occurs and therefore is a 'low-level' mechanism that we first considered in the study. Since adaptation did not fully explain the MEG response, we further considered auditory stream segregation. We agree that our previous writing was confusing and we have now made it clear that (1) adaptation has its own contribution to echo suppression, and (2) the MEG response to echoic speech is better explained when considering auditory stream segregation.

For the first point, it is summarized that:

"Simulation showed that the phase coherence at echo-related frequencies was boosted by neural adaptation for echoic speech, but it remained lower for echoic speech than anechoic speech (Fig 3B), suggesting that neural adaptation could partially restore the envelope of direct sound. Therefore, neural adaptation could possibly explain the neural response at 6 Hz when listening to 0.25-s echoic speech, but could not fully explain the neural responses at 2 and 4 Hz for the 0.25- and 0.125-s echoic speech conditions."

For the second point, we have now incorporated neural adaptation into the TRF

model and demonstrated that combining these two mechanisms best explain the MEG response to echoic speech.

"Additionally, we could also extend the classic linear TRF model by considering neural adaptation (Fig 6A and S4 Fig). The TRF considering neural adaptation significantly outperformed the TRF without neural adaptation in terms of its predictive power (S5 Fig). The relationship between the 3 models, however, remained after considering neural adaptation (Fig 6B-6F). For Experiments 1 and 2, and the first and second conditions of Experiment 3, the adapted streaming model still had the higher predictive power than adapted mixture model (Experiment 1, $p = 0.0019$, Experiment 2, $p < 0.001$, Attending to echoic speech, $p = 0.0096$, Movie watching (echoic speech), $p = 0.0052$, permutation test, FDR corrected) and adapted idealized model ($p < 0.001$ for Experiment 1, Experiment 2, and 1st and 2nd conditions in Experiment 3, permutation test, FDR corrected). For the 3rd condition in Experiment 3, the adapted mixture model still better explained the MEG response than the adapted streaming model ($p < 0.001$, permutation test, FDR corrected) and adapted idealized model ($p < 0.001$, permutation test, FDR corrected)."

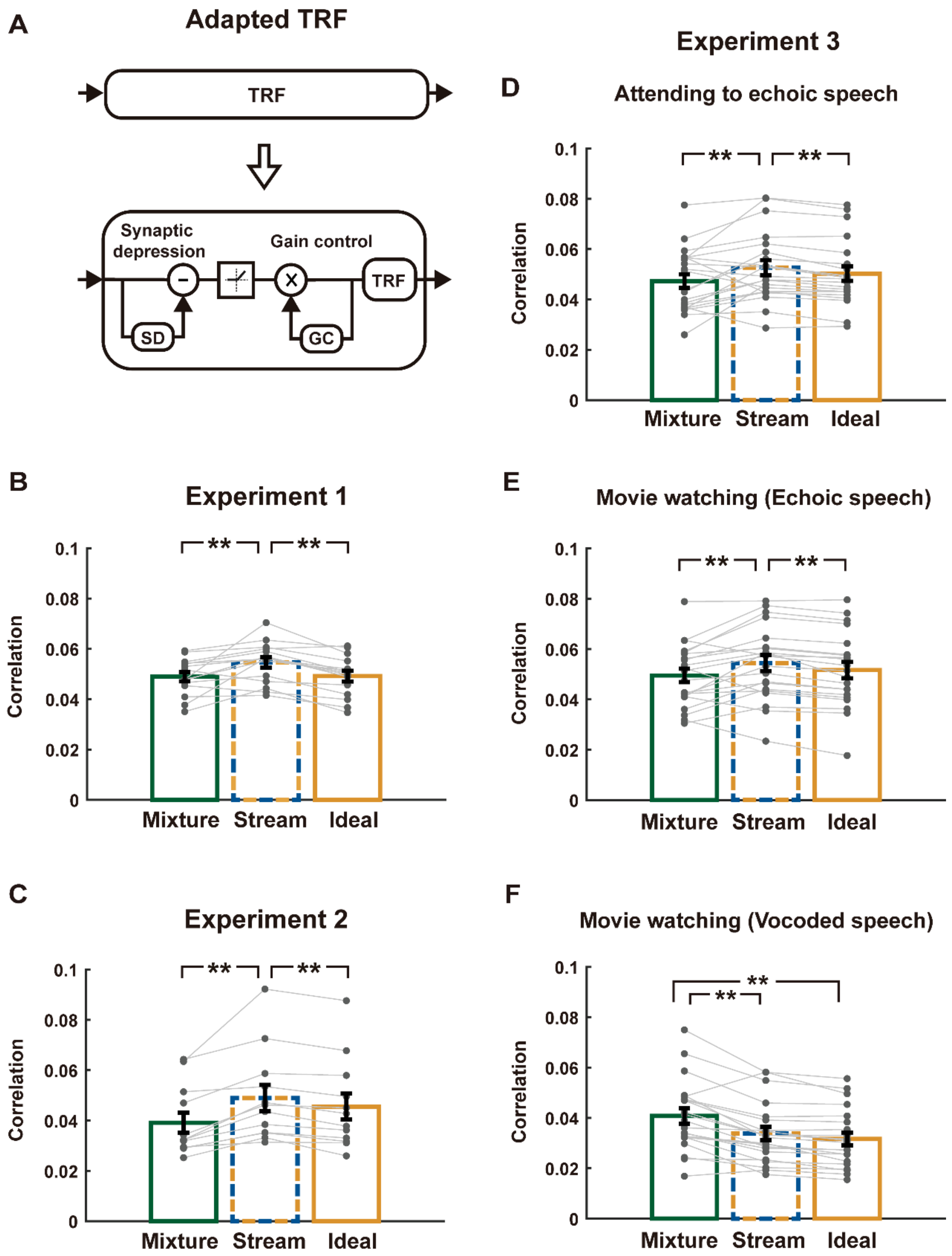

"Fig 6. TRF model based on simulated adapted neural input.

(A) Illustration of the TRF model with adapted neural input – The input to each TRF is adapted based on a computational model (Mesgarani et al., 2014). (B-F) Predictive powers of the TRF models with adapted neural input, averaged over participants and MEG gradiometers. Grey dots show individual participants. Error bars represent 1 SEM across participants."

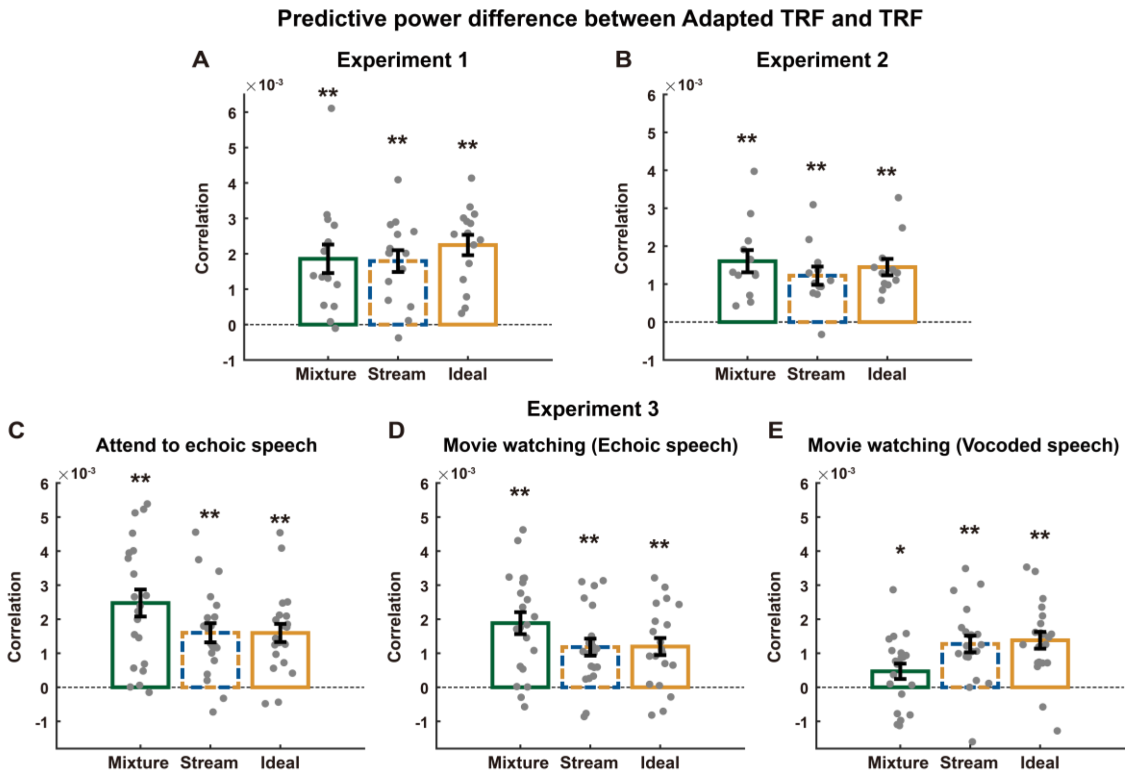

"S5 Fig. Predictive power difference between Adapted TRF and TRF models. The differences between the predictive powers of adapted TRF model and TRF model, averaged over participants and MEG gradiometers. Grey dots show individual participants. Error bars represent 1 SEM across participants."

Minor points:

ll. 131-132: "For speech with a 0.125-s echo, the phase coherence spectrum showed notches at 4 and 12 Hz." Phase coherence is between two signals. The sentence does not tell what was compared with what for the 0.125 echo.

Sorry for this confusion, we have now changed the sentence to:

"The phase coherence spectrum between the simulated neural response and the envelope of direct sound showed notches at 4 and 12 Hz for speech with a 0.125-s echo, and showed notches at 2, 6, 10, and 14 Hz for speech with a 0.25-s echo (Fig 2A)."

II. 245-247: This is the first instance of the reader encountering the term "baseline model", because the methods are described at the end of the ms. A more detailed explanation is needed, telling what is meant by the baseline model (my "Major point" above should also be considered).

Figures 5A and 6A are not very informative as the corresponding information is clearly provided in the main text.

Now we call the model a "mixture model" and have now defined the term when it first appeared.

"The mixture model modeled the MEG response using the temporal envelope of echoic speech, and the idealized model modeled the MEG response using the temporal envelope of direct sound (Fig 4A)."

Figure 6A is removed. Figure 5A may be redundant for readers who are familiar with the TRF analysis, but it provides a straightforward illustration for readers who are not familiar with the TRF analysis. Therefore, we chose to keep this figure.

ll. 455-464: "$s(t)$" and "$As(t)+s(t-\tau)$" carry different energy. Why were the RMS intensities not matched? The possible effects of different energies should be mentioned.

In Experiments 1 and 3, the power of anechoic speech matched the power of the direct sound in the echoic speech, so that if the direct sound could be isolated from the mixture it was fully matched with the anechoic speech, i.e., $s(t)$. Consequently, the anechoic speech and echoic speech were not matched in terms of the total power. We have now discussed the potential influence of sound intensity. Additionally, we have now reported the modulation spectrum, which characterizes the sound intensity at difference modulation frequencies.

"Here, in Experiments 1 and 2, the phase coherence below 2 Hz was generally enhanced for echoic speech than anechoic speech (Fig 2B and 3C, S2 Fig), which might be explained by the increase in sound intensity for echoic speech compared with anechoic speech at low frequencies, as shown in the modulation spectrum (Fig 1B and 1C)."

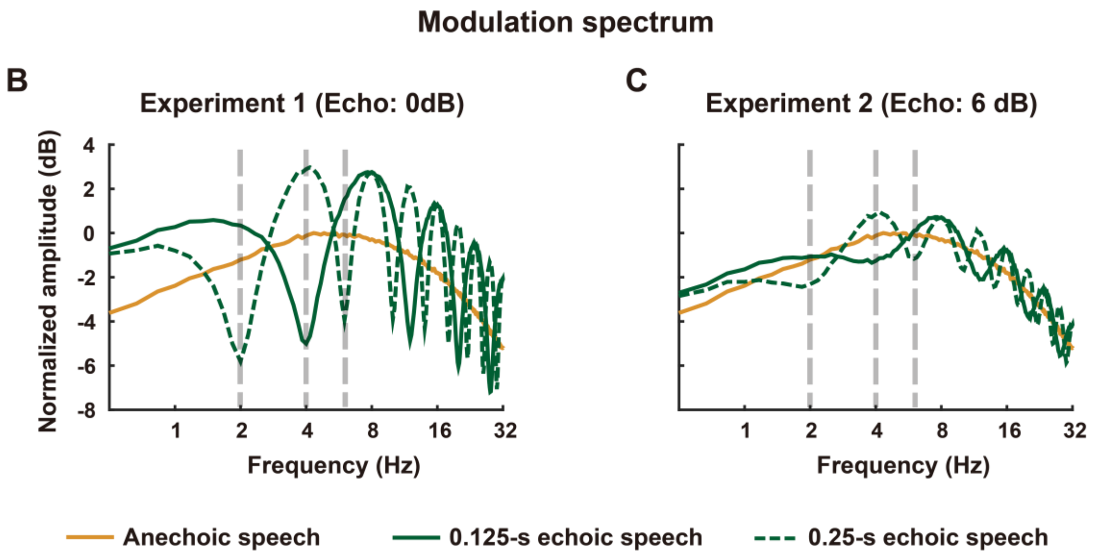

"Fig 1. Construction of echoic speech and the modulation spectrum.
(B) Modulation spectrum of stimulus in Experiment 1. Dashed grey lines denote the echo-related frequencies below 10 Hz. (C) Modulation spectrum of stimulus in Experiment 2, in which the amplitude of the delayed signal is twice the amplitude of the direct sound (6-dB echo)."

ll. 476-481: The description gives the impression that vocoding was applied to the composite (original+echo) signal. If this was the case, then it should be shown that the vocoded signal is still an echoed sound signal. Otherwise, this condition does not test the echo phenomenon. To model how echoes can be resolved for a vocoded signal, the order should be vocoding the original signal than apply echoing.

Sorry for this confusion. We actually used the second method and we have made it clear.

"The 1-channel vocoded speech in Experiment 3 was constructed based on echoic speech with a variable delay. The direct sound and the echo were noise-coded respectively at first, then were mixed to form the echoic vocoded speech."

II. 509-510: Using a subtitled movie for the passive experiments may have caused interference between the texts read (and possibly subvocalized) and the test sounds. This should be noted in the text.

We have now added discussion on this issue.

"The movie and subtitles could also generate neural responses but these responses were uncorrelated with the speech stimulus presented in the experiment. Also, the

processing of subtitles might engage neural pathways that partially overlap with the processing of speech.”

Reviewer #3:

This paper investigates the robustness of human speech perception to the presence of echoes, as occur in some natural environments and as are common in online videoconferencing. Such echoes can distort the amplitude envelope of a speech signal entering the ear, but have little effect on human perception. First, the authors provide evidence that human robustness cannot be explained by adaptation mechanisms previously proposed to explain robustness to reverberation. Second, they show that the envelope of the "direct" speech signal (i.e., without the echo) is represented in the auditory cortex more than would be expected without some compensatory mechanism, as evidenced by phase coherence between the MEG signal and the direct speech envelope. Third, they show that the MEG response to echoic speech is best explained by a TRF model that has two TRFs, one for the direct speech envelope and one for the echo envelope (this does better than a single TRF fit to the envelope of the combined direct+echo speech, or to just the direct speech). This latter result also held for echoic speech in which the echo delay varied over time, and when listeners were watching a movie rather than performing a task with the speech, but was eliminated when the speech was noise-vocoded (a manipulation intended to prevent the direct and echo speech from being streamed). The authors conclude that robustness to echos of this sort is mediated by streaming, with the echo represented as distinct from the direct speech.

My overall assessment is that this is good and novel work. I think the conclusions are supported by the data, and my main suggestions regard the clarity of presentation, which I think can be improved. I am supportive of publication following revisions to address these issues.

Thank you for the accurate summary and positive evaluation.

Issues of interpretation that merit further discussion:

a) I think the authors should make it clear that the "streaming model" that they advocate is a bit different from "generic" streaming (as would happen if someone were listening to two distinct and independent voices). The TRF model they use would not work, I think, if applied to this more generic context. In other words, there is something different about the way the echo is encoded compared to the direct sound, which is why it is possible to use the dual-TRF model in this case. It is perhaps analogous to the situation where one stream is attended and another is not.

Thank you for bringing up this very important issue. On the one hand, previous studies suggest that the model may also work in the more “generic” conditions. A few previous studies have used the method to probe whether a speech mixture is separated into different streams outside the focus of attention [39,51,52]. These studies were cited in the previous manuscript and we have added more discussions. On the other hand, we agree with the analogy with attentive listening – The dual-TRF

model can only work when the two streams are encoded in different manners and we have also added relevant discussions:

"Additionally, the streaming model assumes that the two streams are not just segregated but also encoded in different manners [39,51,52]. When the listener attends to one stream and ignores the other stream, many studies have demonstrated that the two streams are neurally segregated and differentially encoded depending on whether the stream is attended to [13,16,50,53]. Here, since the two streams differed by a time delay, it was possible that the leading stream suppressed the lagging stream through stream-level neural adaptation – For example, when a syllable was recognized from the leading stream, it could potentially adapt the neural response to the same syllable in the lagging stream, leading to differential encoding of the leading and lagging streams. This kind of adaptation occurred together with or after auditory stream segregation and was different from the neural adaptation that only considers the temporal envelope of the auditory stimulus."

b) The results could be taken as evidence that the neural tracking of the speech envelope is better understood as a consequence of speech segregation rather than a cause. Please discuss.

Thank you for raising this very important question. In fact, the same concern also applies to previous studies that investigate speech segregation using MEG or similar techniques. We have added discussions on this point.

"Here, the results suggest that neural activity separately tracks the temporal envelope of the direct sound and the temporal envelope of the echo. The temporal envelope of a sound, however, is extracted even in the auditory periphery, while auditory stream segregation occurs in much later auditory processing stages [3,16,24,48,49], leading to an apparent paradox of why auditory streaming can modulate envelope-tracking activity. A solution to the paradox is the following: What is available in the auditory periphery is the temporal envelope of the mixture, while the temporal envelope of the direct sound or the echo can only be resolved after auditory stream segregation. For example, under the analysis-by-synthesis framework [3,4], the speech mixture is first decomposed into features and features belonging to an auditory stream are selectively grouped together to form a representation of that stream in later processing stages. In other words, the temporal envelope of the sound mixture is extracted in the cochlear while the temporal envelope of an auditory stream is resynthesized in cortex [13,16,50]. Given

the relatively low spatial resolution of MEG and the lack of structural MRI scans from the participants, here we did not investigate where anatomically the temporal envelope of the direct sound is resynthesized. Future studies, possibly requiring intracranial neural recordings from humans or animal neurophysiology, are required to analyze where the segregation between speech and echo emerges along the auditory pathway."

Issues that need to be better explained to the reader:

a) Throughout the paper, it would help to provide more guidance re: the interpretation of results. I would have appreciated being given an explicit conclusion at the end of most paragraphs, confirming my guess as to what the results mean. I have noted a few specific places below. Pages 9 and 10 were particularly tough going - they seemed like a long list of results, with the burden being left on the reader to figure out how they fit together.

Thank you for the valuable suggestion. We have now added brief a summary after each result section.

b) I think you should help the reader think through the consequence of the echo and direct sound being correlated. Please spell out the argument here in more detail, up front. This eventually becomes somewhat clear with the variable delays in Experiment 3, but I think should be addressed head on earlier in the paper.

Thank you for the valuable suggestion. We have added a section in Methods to explain the consequence of having two correlated signals, and referred to this section in Results.

"… r(t) was modeled based on [s_d(t-1), s_d(t-2),…, s_d(t-D), s_e(t-1), s_e(t-2),…, s_e(t-D)] in the streaming model, but was modeled based on just [s_d(t-1), s_d(t-2),…, s_d(t-D)] in the mixture model. If the two input signals, i.e., s_d(t) and s_e(t), were not fully correlated, the streaming model differed from the mixture model in terms of their inputs. Otherwise, e.g., if s_d(t) = s_e(t - d), the input to the streaming model became [s_d(t-1), s_d(t-2),…, s_d(t-D-d)], which reduced to a mixture model."

"In each echoic condition, the direct sound and echo were fully correlated except for a time delay, making it impossible to separate their neural responses using the TRF model (see methods for rationales). Therefore, to dissociate the neural responses to the direct sound and the echo, we pooled stimulus conditions with different echo delays (i.e., 0.125-s and 0.25-s delay) in the TRF analysis."

It is natural for the reader to wonder what happens if you compute phase coherence with the echo rather than the direct sound.

The phase coherence with the echo is the same as the phase coherence with the direct sound, since the phase coherence is insensitive to the delay between signals. We have now mentioned this point.

"In this study, for anechoic speech, we calculated the phase coherence between the MEG response and the speech envelope. For echoic speech, we calculated the phase coherence between the MEG response and the envelope of the direct sound, to characterize whether neural tracking of speech was influenced by the echo. The same result could be obtained when calculating the phase coherence between the MEG response and the envelope of the echo since there is a constant phase lag between the envelope of direct sound and the envelope of echo over time at each frequency."

Specific Comments:

1. The English could use proofreading for grammar (in particular, the use of "the", which seemed to be missing in many places where I would have expected it).

Thank you for the suggestion and we have asked a native English speaker to proofread the manuscript.

2. 100: phase coherence needs to be explained briefly in the main text for a general audience

We have added a brief explanation about phase coherence.

"The phase coherence spectrum quantified the phase locking between two signals in different frequencies. Specifically, the response phase of each signal was extracted in consecutive time windows using the Fourier transform. If the two signals were perfectly synchronized, the phase lag between them would be a constant across all time windows and the phase coherence would reach its maximum, i.e., 1. In contrast, if the two signals were independent of each other, their phase lag would be random and the phase coherence would be at a chance level."

3. 102: unclear - I presume results are averaged across 270 examples, one for each of the 270 IRs? Was the same speech envelope used in each case?

Yes, but the result is now removed.

4. 108: please briefly specified how the parameters were optimized

We have now explained the parameter optimization process in Methods.

"The model contained three parameters, i.e., V, τ_{sd}, and τ_{gc}, which were the firing threshold (0 < V < 1) and the time constants for the synaptic depression and the gain control (0 < τ_{sd} < 500 ms, 0 < τ_{gc} < 500 ms). We tested possible combinations of the parameters (step size = 0.05, 50 ms, and 50 ms, for V, τ_{sd}, and τ_{gc}) and chose the combination to maximize the correlation coefficient between the simulated neural response and the envelope of direct sound that was averaged over the 4 echoic conditions (i.e., 2 delay × 2 experiments)."

5. 132-133: explain for the non-expert why the notches occur where they do

Thank you for the valuable suggestion. We have also added an illustration about why these frequencies are affected by the echo in Fig. 1A.

"Fig 1A illustrates why these notches were created – The Fourier analysis decomposed a signal into sinusoids. If the signal was time shifted by T, forming an echo that had the same amplitude as the direct sound, all its Fourier components would also be shifted by T. Consider a sinusoidal Fourier component whose period was 2T, which had an opposite phase for the original signal and the echo, would get cancelled when the original signal and the echo were mixed. The same applied to Fourier components whose periods were 2T/3, 2T/5, etc. In the following, the frequencies of these sinusoidal components that were notched out by the echo, i.e., 1/2T, 3/2T, 5/2T, were referred to as the echo-related frequencies."

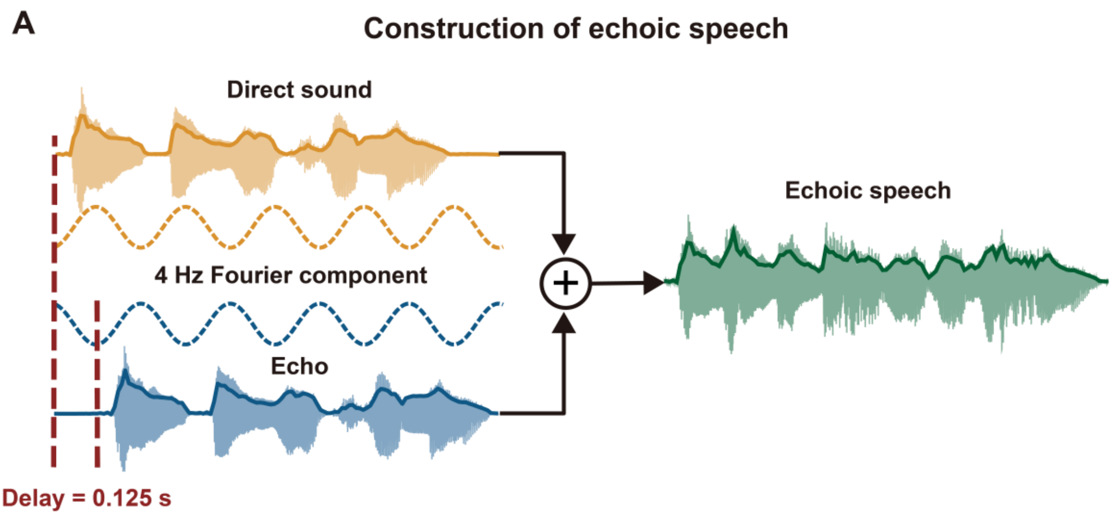

"Fig 1. Construction of echoic speech and the modulation spectrum.
(A) In Experiment 1, the echoic speech is generated by delaying a speech signal by T (0.125 s or 0.25 s) and adding together the delayed signal with the original signal. The dashed sinusoids in yellow and blue represent the 1/2T Hz Fourier components of the envelope of direct sound and echo, respectively. The two sinusoids are 180 degrees out of phase and are fully cancelled in the mixture."

6. 151: please spell out the conclusion, rather than leaving it implicit

The section is now removed. However, in the new section on neural adaptation, we have explicitly summarized the interpretation.

"Simulation showed that the phase coherence at echo-related frequencies was boosted by neural adaptation for echoic speech, but it remained lower for echoic speech than anechoic speech (Fig 3B), suggesting that neural adaptation could partially restore the envelope of direct sound. Therefore, neural adaptation could possibly explain the neural response at 6 Hz when listening to 0.25-s echoic speech, but could not fully explain the neural responses at 2 and 4 Hz for the 0.25- and 0.125-s echoic speech conditions."

"Taken together, consistent with Experiment 1, neural adaptation could possibly explain the neural response in Experiment 2 at 6 Hz, but could not fully explain the neural response at 2 and 4 Hz"

7. 164: "comprehensions" is the wrong word

It should be "comprehension questions".

8. 172: please state the conclusion we should draw from this set of results

We have added a summary.

"Within this frequency range, i.e., below 10 Hz, we would probe whether the MEG activity can track the speech envelope at echo-related frequencies when the participants listening to echoic speech."

9. 195-198: Is this result predicted by some hypothesis? How are we to interpret it? Please tell me what I should think about it.

These results are not related to our hypothesis and are now removed. However, we do discuss the potential cause for the phenomenon in Discussion.

"Here, in Experiments 1 and 2, the phase coherence below 2 Hz was generally enhanced for echoic speech than anechoic speech (Fig 2B and 3C, S2 Fig), which might be explained by the increase in sound intensity for echoic speech compared with anechoic speech at low frequencies, as shown in the modulation spectrum (Fig 1B and 1C)."

10. 218: please state the conclusion we should draw from this set of results

Please see the response to question #6.

11. Figures 3 and 4 should be reversed in order, in my opinion, to create parallel organization of the results of experiments 1 and 2.

We have modified the figures and the two figures about Experiments 1 and 2 are now next to each other.

12. 264: please spell out the logic of the argument for why the TRF analysis provides evidence for segregation into separate streams

We have now made the rationales clear.

"The fact that the streaming model outperformed the two alternative models suggested that the auditory system segregated the direct sound and the echo into separate streams and encoded them differently."

13. 270: the word "barely" was confusing to me. 273: same here

We have changed it into "not".

14. 305: please give the conclusion the reader should draw from these results

We have added a summary.

"In this condition, the mixture model better explained the MEG response than the streaming model (Fig 5C, left plot, p < 0.001, permutation test, FDR corrected) and the idealized model (Fig 5C, left plot, p < 0.001, permutation test, FDR corrected), suggesting that the stimulus was encoded as a whole instead of two separate streams."

15. 49: I think it would make sense to acknowledge that such echoes do sometimes occur in natural environments, and that this could ultimately be the reason why we are able to handle echoes in this way. It at least seems like a plausible possibility. I don't think the results are exclusively relevant to Zoom…

We agree and we have now mentioned that echoes do sometimes occur in natural environments.

"Echoes are perceptually salient acoustic phenomena. For example, it has been suggested that ancient rock art is often created at places that can generate maximal echo intensities [42], and there are a number of places that are famous for generating echoes, such as the echo wall at the Temple of Heaven in Beijing and the whispering gallery of St Paul's Cathedral in London."

16. 366-368: this idea should be explained earlier, when you show the initial modeling results

We have now explained the idea when introducing Experiment 2.

"Experiment 2 was the same as Experiment 1 except that the echo was 6 dB more intense than the direct sound. Simulations showed that the neural adaptation aggravated, instead of alleviating, the loss of speech envelope at echo-related frequencies in Experiment 2 (Fig 3B)."

17. 394: reference 34 should be mentioned in the introduction

The reference was actually mentioned in the introduction but we have reorganized the introduction to make it clearer.

"Furthermore, evidence has been provided that a click and its echo would be encoded and perceived as separate auditory objects [34]. It remains unclear, however, whether these mechanisms can restore the temporal modulations eliminated by echoes."

Reviewer #4:

In this article by Gao et al. the neural tracking of speech envelope in the presence of a single, long-delay echo is investigated using magnetoencephalography. Potentially, this article may be an interesting addition to the growing number of studies that investigated continuous speech streams using some form of MEG, EEG, ECoG-based tracking. The article put forward the hypothesis that, in the presence of a single echo, the direct speech and the echo are automatically segregated in two streams in the auditory cortex. This is a suggestive and interesting hypothesis, however, I am not convinced that the evidence provided is sufficiently strong to convincingly support it. As described below in detail, I find that

1) not all results are actually consistent with this hypothesis,

2) currently unexplained effects may be more relevant than those examined and that

3) the statistical approach can be improved and should be more conservative.

Below are my specific comments:

The first part of the results section (Figure 1A, 1B, 1C) describes an analysis of the stimuli showing that the echo creates phase distortions to the speech envelope at specific temporal modulation frequencies (related to the echo delay). Based on this observation, the authors postulate their working hypothesis that successful neural tracking of missing echo-related components provides evidence for auditory stream segregation of the directed and shifted speech stream.

I find some of the results in Figure 1 unintuitive: In Figure 1C, for instance, phase distortion is larger when the echo has the same amplitude as the direct sound (Exp. 1) than when the echo is twice stronger than the direct sound (Exp.2). What is the reason for this? May this reflect somehow the overall energy/loudness of the stimuli? Were anechoic and echoic speech normalized in energy/loudness? How? (In Figure 1D, the pattern of results for the neural adaptation simulation is more like one would expect, with stronger effects for the more intense echo signal).

Thank you for pointing out this issue. First, we have now modified Fig. 1 to explain why the echo most strongly interfere with the direct sound when they have equal amplitude.

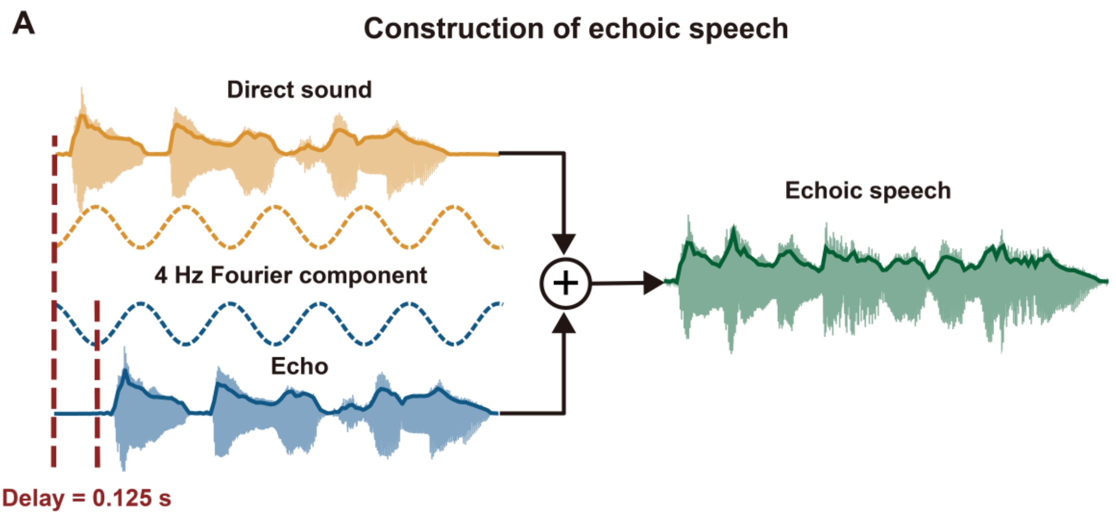

"Fig. 1. Construction of echoic speech and its modulation spectrum.
(A) In Experiment 1, Echoic speech is generated by delaying a speech signal by T (0.125 s or 0.25 s) and adding together the delayed signal the original signal. The dashed sinusoids in yellow and blue represent the 1/2T Hz Fourier components of the envelope of direct sound and echo, respectively. The two sinusoids are 180 degrees out of phase and are fully cancelled in the mixture."

We can also consider an extreme case. Suppose the echo is so strong compared with the direct sound so that the direct sound can be ignored. In this condition, the echoic speech reduces to the echo, which shares all properties of anechoic speech except for a delay.

Second, the anechoic and echoic speech are not normalized in loudness so that the direct sound in echoic speech is the same as the anechoic speech in Experiments 1 and 3. We have now made it clear in Methods and discussed the potential influence of sound intensity.

"In Experiments 1 and 3, the echoic speech was 3.01 dB stronger than the anechoic speech in terms of RMS of the sound waveform. In Experiment 2, the echoic speech was 0.97 dB stronger than the anechoic speech."

"Here, in Experiments 1 and 2, the phase coherence below 2 Hz was generally enhanced for echoic speech than anechoic speech (Fig 2B and 3C, S2 Fig), which might be explained by the increase in sound intensity for echoic speech compared with anechoic speech at low frequencies, as shown in the modulation spectrum (Fig 1B and 1C)."

This first part also includes an additional analysis conducted to exclude the possibility that neural adaptation mechanisms in the auditory system would compensate these echo-related distortions, the authors simulated (with several models) the neural responses with adaptation mechanisms to the echoic (and anechoic) speech and calculated their coherence them with the anechoic speech envelope. Results showed a reduced phase coherence between simulated neural responses and echoic speech envelope at the echo-related modulation frequencies. These results are taken as evidence that neural adaptation mechanisms are not sufficient to restore phase coherence in the presence of echoes and an alternative mechanism is required.

While I appreciate the potential relevance of this analysis and the need to exclude obvious alternative hypotheses, I am not sure that this is the right place for this description. Without first reading the MEG results, I missed the message this analysis wants to convey. However, it became clearer after a second reading of the paper. I would consider moving them later in the paper or, alternatively, in the supplementary materials.

Thank you for the valuable suggestion. We have now greatly simplified the section on neural adaptation and used it to introduce Experiment 2.

Figure 2 shows the results of the MEG tracking of anechoic and echoic (Exp.1, 0 dB, 0.125 s and 0.25 s) speech on the phase coherence at different temporal modulations. Here the authors focus on the lack of differences between the tracking of echoic and anechoic speech at the echo-related modulation frequencies support to their hypothesis with ad hoc statistical tests (Figure 3, see below my comments on the statistical analysis). These tests confirmed the hypothesis at 4 Hz for 0.125 s delay, and at 2 Hz for 0.25 s, but not at 6 Hz for 0.25 s.

Sorry for the confusion. We have now clearly stated our hypotheses and conclusions.

"*Our hypothesis, referred to as the envelope restoration hypothesis, was that the auditory system could fully or partially restore the temporal envelope of direct sound, and an alternative hypothesis was that the auditory system faithfully followed the temporal envelope of the echoic speech. We first quantified the prediction of the alternative hypothesis through a simulation... Therefore, if the phase coherence between the neural response to echoic speech and the temporal envelope of direct sound is 0 at echo-related frequencies, the alternative hypothesis is supported. Otherwise, the envelope restoration hypothesis is supported and full restoration is suggested if the phase coherence value is comparable for the neural responses to echoic speech and direct sound.*"

"The current experiment also suggested that, in an echoic environment, the auditory system could more reliably restore the very low-frequency components of the speech envelope compared with the higher-frequency components. In Experiment 1, the speech envelope was fully restored at 2 and 4 Hz but was only partially restored at 6 Hz (Fig 2B). Similarly, in Experiment 2, the speech envelope was fully restored at 2 Hz, partially restored at 4 Hz, and not restored at 6 Hz (Fig 3C)."

I was surprised that the authors give little (or no) attention to what seems to be a major effect of the echo in Figure 2: an enhanced phase coherence at the low modulation frequencies. What is the interpretation of this effect? I feel that if this point is not addressed convincingly, it becomes difficult shifting the readers' focus on the much smaller echo-related effects. This is not addressed in the Discussion either.

Thank you for raising this point. We have now added discussions about why the low-frequency response was enhanced.

"Here, in Experiments 1 and 2, the phase coherence below 2 Hz was generally enhanced for echoic speech than anechoic speech (Fig 2B and 3C, S2 Fig), which might be explained by the increase in sound intensity for echoic speech compared with anechoic speech at low frequencies, as shown in the modulation spectrum (Fig 1B and 1C)."

My reading of the results of Experiment 2 (in Figure 4) is that they are only partially consistent with those of Experiment 1 and with the hypothesized mechanisms. In two out of the three relevant tests (i.e. 4Hz for 0.125 and 6 Hz for 0.25s) there is significant reduction of phase locking compared with the responses to the anechoic speech.

In response to a previous question, we have now clearly stated that our basic hypothesis is about whether the phase coherence at echo-related frequencies is zero. Experiment 2 was designed to minimize the contribution of adaptation on neural restoration of speech envelope. Consequently, as the reviewer correctly pointed out, phase coherence at 4 and 6 Hz are reduced. We have now more clearly explained the purpose and results of Experiment 2.

"Attenuating the neural response to the echo, however, did not always have a positive effect on restoring the envelope of direct sound. For example, if the echo was stronger than the direct sound (Fig 3A), attenuating the echo response could make the neural responses to echo and the direct sound had a more similar

amplitude, cancelling the temporal envelope at echo-related frequencies. Motivated by this idea, we constructed Experiment 2, in which the echo was more intense than the direct sound, to further probe whether neural restoration of speech envelope could be well explained by neural adaptation. If neural adaptation played a dominant role in restoring the speech envelope, the phase coherence between the neural response to echoic speech and the temporal envelope of direct sound should significantly reduce at echo-related frequencies compared with Experiment 1.”

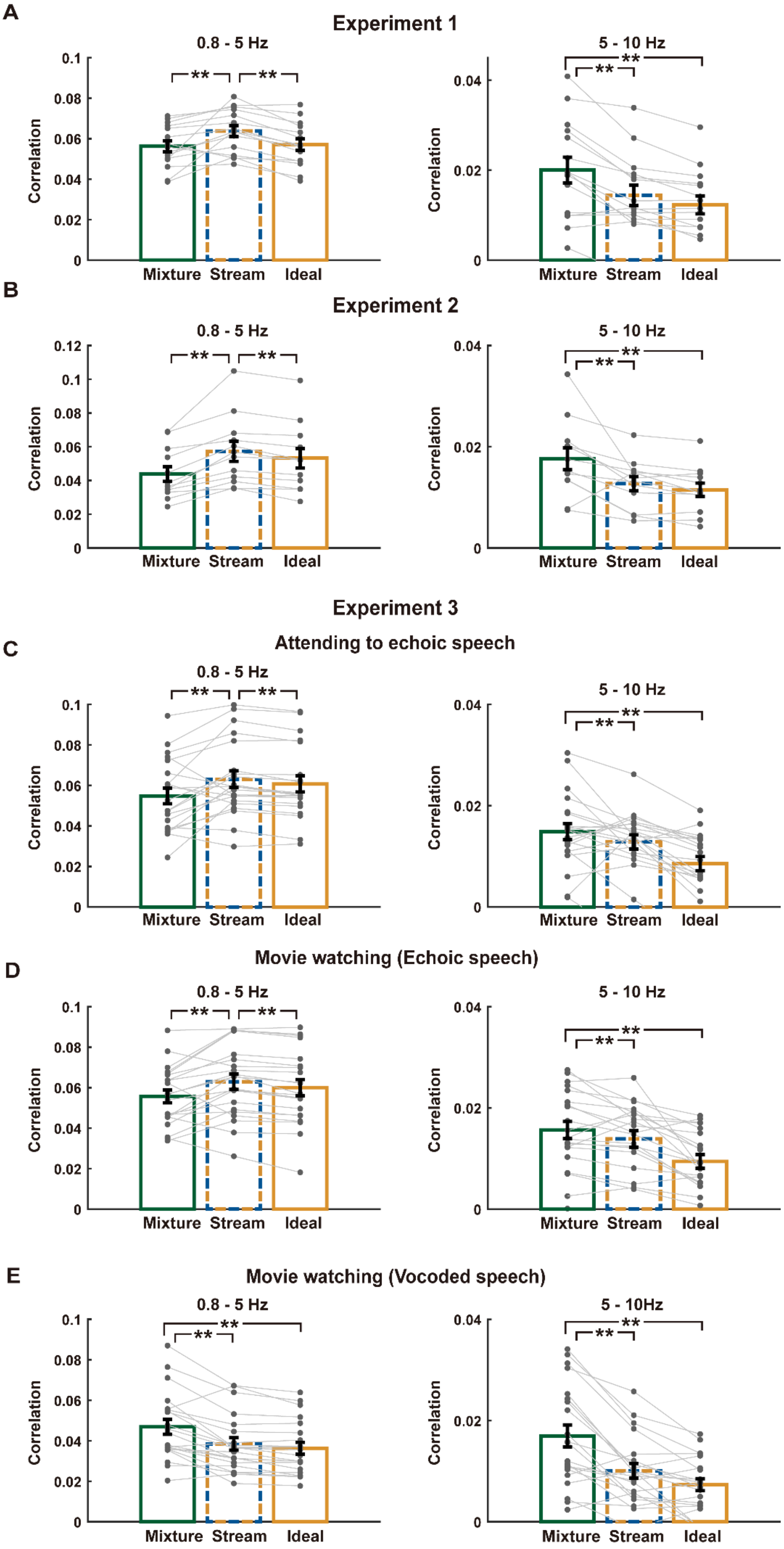

A
Experiment 1
0.8 - 5 Hz
5 - 10 Hz
Correlation
Mixture
Stream
Ideal
B
Experiment 2
Experiment 3
C
Attending to echoic speech
D
Movie watching (Echoic speech)
E
Movie watching (Vocoded speech)
5 - 10Hz

"S3 Fig. TRF models in 0.8-5 Hz and 5-10 Hz frequency bands.

Predictive powers of TRF models in 0.8-5Hz (left plots) and 5-10 Hz (right plots), averaged over participants and MEG gradiometers. Grey dots show individual participants. Error bars represent 1 SEM across participants."

Additionally, I am not convinced of the robustness of the performed statistical analyses. If I understood correctly the actual implementation, the bootstrap procedure implemented to perform the two-sided paired comparison may be quite liberal, corresponding to a fixed-effects comparison. The authors could perform a random-effects non-parametric test by comparing the observed group average difference to a null distribution obtained by re-computing the group average difference after swapping the condition for a randomly selected subset of subjects ($2^{\text{N subjects}}$ number of permutations possible).Overall, for what concerns the analysis of modulation-specific phase coherence, I do not find that the evidence provided to support the formulated hypothesis is compelling, both because of inconsistencies in the observed patterns of results and because there seem to be other bigger effects that may be more relevant than those examined. In addition, I think that the statistical approach needs to be clarified and possibly revised.

Thank you for the valuable suggestion, and we have now changed the bootstrap test to the suggested random-effects non-parametric test. We have also shown individual data on Fig. 2C and 3D, and uploaded the analysis scripts.

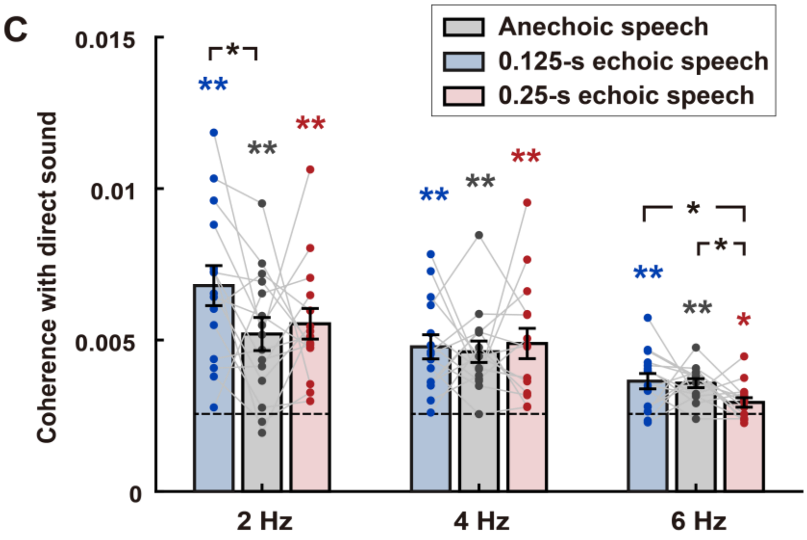

"Fig 2. Results of Experiment 1.

(C) Phase coherence at echo-related frequencies. Each dot represents one individual and error bars represent 1 SEM. Dashed black lines show chance-level phase coherence. Phase coherence significantly higher than chance level and significant differences between conditions are marked (* p < 0.05, ** p < 0.01, permutation test, FDR corrected)."

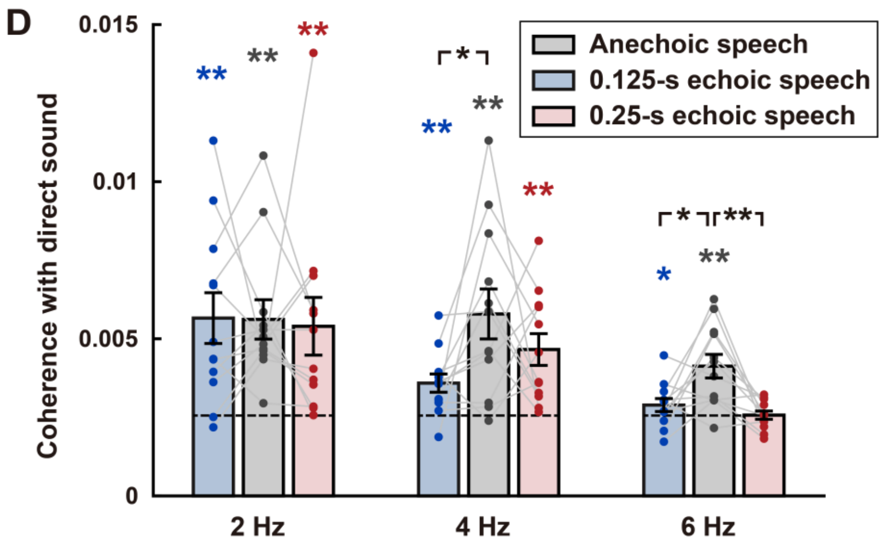

"Fig 3. Results of Experiment 2.
(D) Phase coherence at echo-related frequencies of Experiment 2 is shown with the same conventions in Fig. 2C."

To further support the hypothesis that measured MEG responses reflect the auditory streaming of direct sound and echo the authors conducted a TRF analyses, comparing a baseline (echoic speech), streaming (direct sound, echo) and idealized (direct sound) models. In line with the streaming hypothesis, results reported in Figure 5 seem to indicate that the two predictors model predict is (slightly) better than the other models. Results from Experiment 3 (Figure 6) with variable delays confirm that the streaming model has a slight higher predictive power except in the case where relevant speech cues have been removed (vocoded speech). In principle, this is, in my view, the strongest evidence the authors in support of the streaming hypothesis. However, the description of the methods employed does not allow judging whether the conclusions are sound. In particular, I am missing two aspects:

1. A precise description on how the data for model fitting and estimation of the predictive power is separated (e.g. at which level of the processing pipeline). This is important to assess to what extend fitting and testing data can be considered independent.

Sorry for missing these details. They are now added to the method section:

"The TRF was independently computed for each model and each participant using ridge regression [65]. The predictive power of a model was defined as the correlation between the actual MEG response and the TRF prediction. The model was evaluated using 10-fold cross-validation. Specifically, each participant's MEG response was evenly divided into 10 segments. Nine segments were used to train the model, and the remaining segment was used to evaluate the predictive power of the model. The

10-fold cross-validation procedure resulted in 10 estimates of averaged predictive power and TRF. The regularization parameter for ridge regression was separately optimized for each model and each experimental condition."

2. A precise description on how model differences is assessed statistically. As a specific description is missing, I am assuming that this is done as in the phase coherence analysis. Thus, my previous comment on the need of implementing random-effects statistics applies also in this case.

Yes, it was analyzed the same way as in the phase coherence analysis. Now, following your valuable suggestion, we have now employed a random-effects non-parametric test.

"When comparing the phase coherence or the TRF predictive power across conditions, we performed a permutation test, in which the condition label was switched for a subset of participants before calculating the mean difference across conditions. To obtain the null distribution of the mean difference across conditions, the full set of 2^N possible permutations of N participants was considered. Here, we applied a one-sided test to evaluate, e.g., whether the phase coherence is higher when listening to the anechoic speech than the echoic speech, and whether the streaming model has a higher predictive power than the alternative models. If the actual difference across conditions (mean over participants) was smaller than M out of the 2^N permutations, the significant level was $(M + 1)/(2^N + 1)$."

Finally, there is quite some discrepancy between the shape of the TRFs for Experiment 1 and 2, almost with a switch between signals. This may not be necessarily a problem, but I think it deserves to be discussed. In Experiment 3 TRFs are more consistent but also in this case it would be interesting to discuss the shape of the obtained TRFs.

The shape of the TRF indeed deserved more discussion. The most dramatic difference between the TRFs in Experiments 1 and 2 is the relative response gain for the direct sound and the echo, and we have added a discussion about this phenomenon, as well as the shape of the TRF in Experiment 3.

"The TRF for the echo had higher amplitude than the TRF for the direct sound in Experiment 1 but the pattern was reversed in Experiment 2. This result suggested that although the gain of the echo was increased in the stimulus of Experiment 2, the neural response gain for the echo was reduced."

In Experiment 3,

"The shape of TRFs in these conditions was similar to that in Experiment 1, in which the direct sound and the echo also had equal amplitude."
